# Measurement of interscalene space volume in diagnosis of thoracic outlet syndrome: a cadaver study

**DOI:** 10.3906/sag-2101-375

**Published:** 2021-08-30

**Authors:** Tevfik KAPLAN, Ayhan CÖMERT, Mehmet Ali GÜNER, Halil İbrahim AÇAR, Gökçe Kaan ATAÇ, İbrahim TEKDEMIR, Serdar HAN

**Affiliations:** 1 Department of Thoracic Surgery, Lokman Hekim University Hospital, Ankara Turkey; 2 Department of Anatomy, Ankara University School of Medicine, Ankara Turkey; 3 Department of Radiology, Ufuk University School of Medicine, Ankara Turkey

**Keywords:** Thoracic outlet syndrome, surgery, diagnosis, anatomy, cadaver

## Abstract

**Background/aim:**

The aim of this study was to measure the volume of interscalene space in thoracic outlet region on cadavers and radiological images and to analyze the potential value of these measurements in diagnosis and treatment of thoracic outlet syndrome (TOS).

**Materials and methods:**

The dimensions of the anterior interscalene space in 8 formalin-fixed human cadavers were studied by direct measurement and additionally evaluation of the volume of this space were done by using mold and volume calculation formula of square pyramid, due to resembling a pyramid. In the second phase of this study, interscalene space volume was calculated by formula and compared to calculations from computed tomography (CT) sections in 18 TOS and 16 control patients.

**Results:**

There was a strong correlation between the volume calculated by formula (4.79 ± 2.18 cm^3^) and by mold (4.84 ± 1.58 cm^3^), (R = 0.934, p = 0.001) in cadavers. The average volume measured in TOS patients (2.05 ± 0.32 cm^3^) was significantly smaller than control patients (4.30 ± 1.85 cm^3^, p < 0.0001). There were excellent or good results in 14 patients whereas in 4 patients who had neurogenic TOS achieved fair results after surgery. In these 4 patients the average volumes of abnormal sides were close to the healthy sides.

**Conclusion:**

In our study, volume of interscalene space in TOS patients was statistically smaller than control group. Also, the volume was even smaller in patients with excellent or good results after surgery. In this respect, volumetric measurements from CT sections could be used in diagnosis and treatment selection in TOS patients.

## 1. Introduction

Thoracic outlet syndrome represents a complex of signs and symptoms due to compression of neurovascular structures in cervico-thoracic area. Three anatomical locations responsible for the compression of neurovascular structures include inter-scalene space, costo-clavicular space, and retro-pectoralis minor space [1]. Various abnormalities originating from ribs or scalene muscles and trauma to this area can narrow these spaces and results in compression of neurovascular structures. There are 3 different types of thoracic outlet syndrome (TOS), which are classified according to the trapped anatomical structure and clinical symptoms resulting in neurogenic, arterial and venous TOS[2]. Neurogenic TOS composes approximately 90% of TOS cases. It is more difficult to diagnose since there is no definitive neurodiagnostic testing and objective criteria for the diagnosis of TOS[3]. 

In spite of the treatment of patients with arterial or venous TOS is clear, treatment of patients with neurogenic TOS is the subject of continuing controversy. Surgical results vary, with patients reporting good or excellent results in 53% to 92% of cases[4,5]. 

In this study, the volumetric measurement of interscalene space was done in cadavers and this information was then transferred to clinical situation of TOS and the data were analyzed for the potential use in diagnosis and treatment selection.

## 2. Materials and methods

### 2.1. The first phase of the study

**Figure 1:**
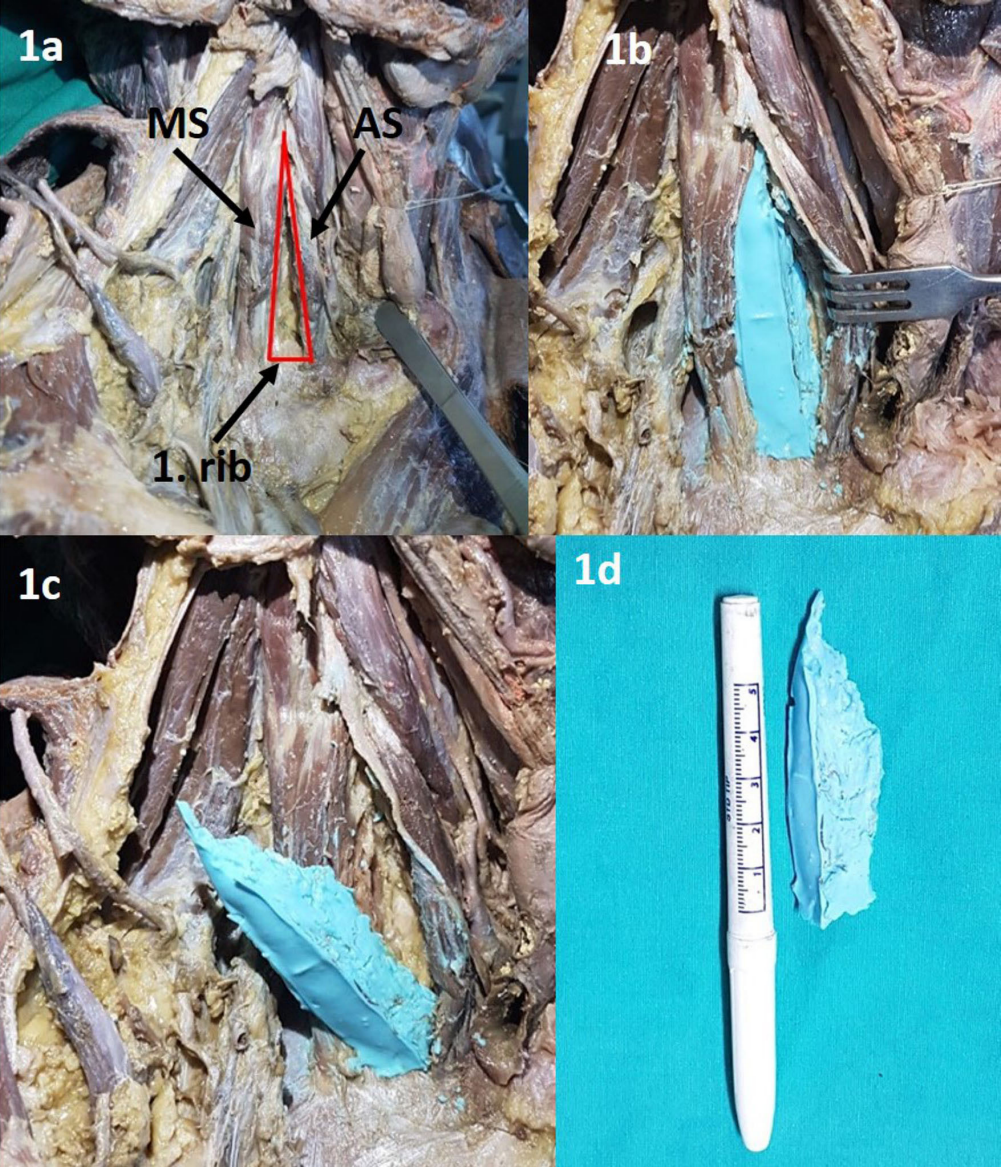
a) Interscalene space after removing all anatomical structures in it. MS: Middle scalene, AS: Anterior scalene. 1b) The mold of the potential cavity between the interscalene muscles was created with alginate material. 1c) Removing the mold of interscalene space. 1d) The mold of the interscalene space.

In the first phase of the study 16 cervical dissections were performed in 8 formalin-fixed cadavers. Dissections were made in supine anatomical position of cadavers. First an incision was made from the thyroid cartilage to the body of the sternum along the midline. Then the incision was extended on both sides at the base of the neck. Skin flaps and platysma were removed. The distal insertions of sternocleidomastoid and pectoralis major muscles were detached and elevated. After elevating the medial end of the clavicle, the dissection was made for removing all anatomical structures in the interscalene triangle (Figure 1a). After morphological measurement of interscalene triangle, the mold of the potential cavity between the interscalene muscles was created with alginate material (Figure 1b-1c) and the volume of the removed mold was measured by water overflow method (Figure 1d).

Alginate is a commonly used measurement material for anatomical modeling. The essential reagent in its content is the sodium and potassium salt of alginic acid and forms a cross-linking with water to form a gel. These substances are used in many areas of dentistry. There are two types of alginate material. Type 1 alginate solidifies in about 2 min, while solidifying time in type 2 alginates is much longer[6]. In this study, type 1 fast solidifying (BLUEPRINT Dust-Free Alginat; DENTSPLY DETREY GmbH GERMANAY) alginate was used. 

**Figure 2:**
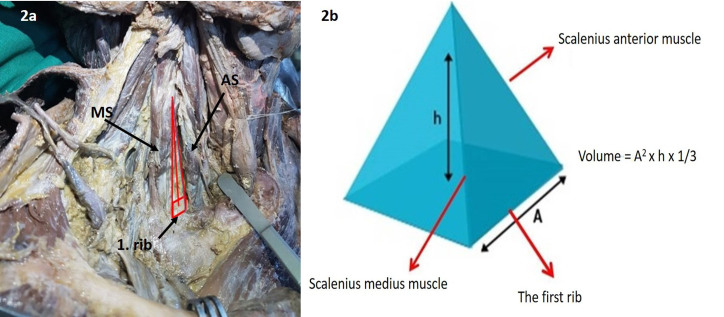
a) The mold of interscalene space, resembles a square pyramid. MS: Middle scalene, AS: Anterior scalene. 2b) The volume formula of square pyramid. A: Interscalene triangle base length, h: Interscalene triangle height.

In addition, since the interscalene space, which is molded by alginate, resembles a square pyramid (Figure 2a-b), so the volume of this cavity was calculated by the volume calculation formula of the square pyramid (the base length of the interscalene triangle^2^ x the height of interscalene triangle x 1/3). All measurements in cadavers were done by 2 anatomists and one thoracic surgeon. A digital caliper used for morphometric measurements in cadavers. Correlation between the calculated volume and the measured volume was evaluated by the Spearman correlation test. A strong correlation was found between the volume calculated by the formula and the volume measured by alginate (R = 0.934, p = 0.001).

###  2.2. The second phase of the study.

**Figure 3:**
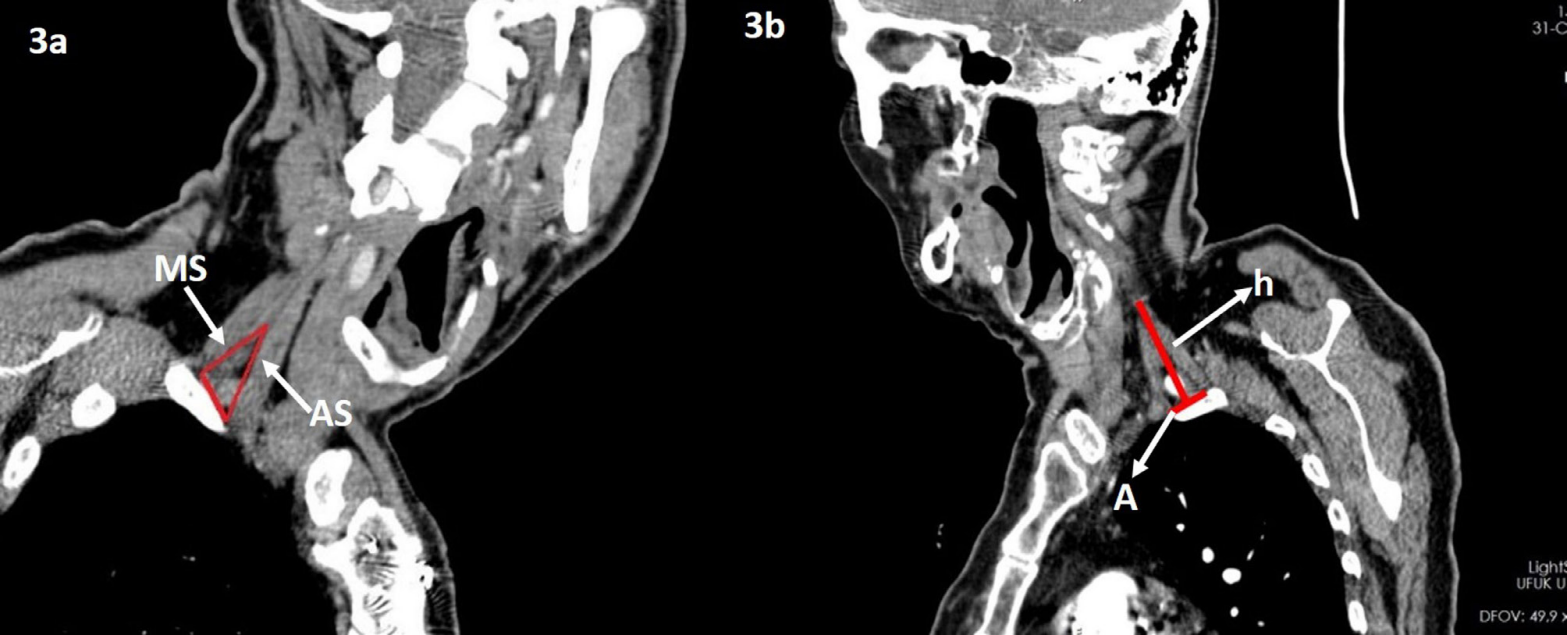
Thoracic CT sections of patients who were operated with the diagnosis of TOS. MS: Middle scalene, AS: Anterior scalene, h: Interscalene triangle height, A: Interscalene triangle base length.

In the second phase of the study, neck and upper thoracic CT sections of 18 patients who were operated with the diagnosis of TOS were retrospectively evaluated (Figure 3a-b). As a control group the CT sections of 16 patients who underwent carotid artery CT angiography (CTA) for a diagnosis other than TOS were examined retrospectively. The measured values ​​of patients with TOS were compared with the measured values ​​from the patients in the control group.

All CT studies were done with a 16-detector row CT scanner (GE Lightspeed 16, General Electric Health Systems, Milwaukee, WI, USA). The patients were in supine and anatomical position. Images of all studies were collected from a picture archiving and communication system (PACS, Clearcanvas, Synaptive Medical, Toronto, ON, Canada). Multiplanar reconstructions with oblique planes were designed manually to identify the dimensions of interscalene space. This procedure does not require a significant amount of time and training. 

An experienced radiologist performed the measurements from the CT sections on 3 different sessions. CT sections of those neck CTA studies were 1.25 mm in thickness with pitch value of 1 and beginning from aortic arch to the circle of Willis region. CT sections performed with contrast media follow up by technologist to see the optimal contrast abundance in vascular structures. All CT sections obtained after two perpendicular localizing images and 120 kVp with optimum tube current regulated by tube current modulation.

The morphometric measurements of interscalene triangle were done in both TOS patients and control group. Also, the volumetric measurements of interscalene spaces were calculated by the square pyramid volume calculation formula. The measured values ​​of patients with TOS were compared with the measured values ​​from the patients in the control group,

### 2.3. Statistical analyses

 For the continuous data Shapiro–Wilk’s test was used for assessing the normality of the data. If the data, follow normal distribution we used parametric test (t-test) for analyzing the differences between the patient and control groups. Homogeneity of variances was also evaluated by Levene test. Comparison of differences between calculated volumes of cadaveric and CT measurements were also performed. We used Mann–Whitney-U test as nonparametric test for comparing the left and right-side measurements of cadavers because of small sample size. P value < 0.05 was considered statistically significant. Spearman correlation test was used to assess the correlation between the calculated volume and the measured volume of interscalene triangle in cadavers. 

## 3. Results

### 3.1. The first phase of the study

The average age of the cadavers was 73.68 ± 6.7 years, and the male/female ratio was 5/3. Interscalene triangle base length, interscalene triangle height, calculated interscalene space volume by formula and volume measured by alginate were 19.01 ± 2.93 mm, 54.77 ± 6.11 mm 4.79 ± 2.18 cm^3^, and 4.84 ± 1.58 cm^3^, respectively (Table 1). There was not statistically difference between the measurements of the right and left sides of the cadavers (Table 2). There was strong correlation between the volume calculated by the formula and the volume measured by alginate (R = 0.934, p = 0.001). In addition, we also found a strong correlation between interscalene triangle height (R = 0.810, p = 0.015) and interscalene triangle base length (R = 0.952, p < 0.0001) with interscalene volume. These results show that the volume of the interscalene space can be calculated by the volume calculation formula of the square pyramid.

**Table 1:** Measurements done from cadavers and the control patients.

	Cadavers	Control patients	p
	n = 16(mean ± SD)	n = 32(mean ± SD)	
Interscalene triangle base length (mm)	19.01 ± 2.93	17.10 ± 5.45	0.51
Interscalene triangle height (mm)	54.77 ± 6.11	47.69 ± 6.30	0.75
Interscalene space volume by formula (cm³)	4.79 ± 2.18	4.30 ± 1.85	0.18

**Table 2:** Measurement of thoracic outlet region in cadavers.

N = 8	Left(mean ± SD)	Right(mean ± SD)	p
Interscalene triangle base length (mm)	19.33 ± 3.24	18.69 ± 2.63	0.12
Interscalene triangle height (mm)	53.72 ± 5.23	55.82 ± 6.98	0.43
Lateral edge of interscalene triangle (mm)	66.11 ± 10.25	66.49 ± 10.90	0.13
Medial edge of interscalene triangle (mm)	66.08 ± 5.72	68.37 ± 6.32	0.75
Top angle of the interscalene triangle (0)	12.75 ± 5.44	14.87 ± 5.54	0.89
Interscalene space volume by alginate (cm3)	4.85 ± 1.70	4.82 ± 1.45	0.38
Interscalene space volume by formula (cm3)	4.62 ± 2.32	4.95 ± 2.04	0.17

### 3.2. The second phase of the study

The average age of the control patients whose CT angiography images were used was 56.45 ± 11.96 years and the male/female ratio was 9/7. In the control group the interscalene triangle base length, interscalene triangle height and interscalene space volume were 17.10 ± 5.45 mm, 47.69 ± 6.30 mm, and 4.30 ± 1.85 cm^3 ^respectively (Table 1). There was no statistically significant difference between the measurements (interscalene triangle base length, interscalene triangle height and interscalene space volume) of cadavers and control patients’ tomographic sections. 

**Figure 4:**
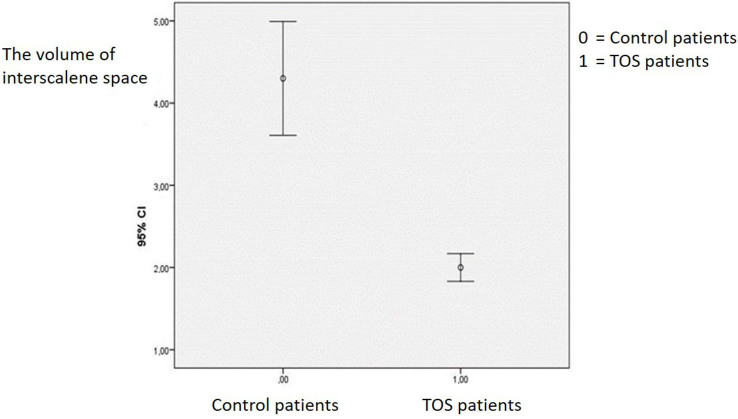
Interscalene space volume were significantly narrower than the control patients.

In TOS patients group the average age was 44.38 ± 7.16 years and the male/female ratio was 8/10. There were 13 neurogenic, 5 arterial TOS patients in the group. In 2 patients there were bilateral TOS, so measurements were done in 20 interscalene triangles. In TOS patients’ group, the interscalene triangle base length, interscalene triangle height and interscalene space volume were 12.49 ± 0.78 mm, 35.46 ± 4.13 mm, and 2.05 ± 0.32 cm^3 ^respectively (Table 3). In TOS patients the measurements of interscalene triangle base length, interscalene triangle height and interscalene space volume in the abnormal side were statistically narrower than the normal side (p = 0.001) (Table 4). Also in TOS patients, interscalene base length, interscalene triangle height, and interscalene space volume were significantly narrower than the control patients (p < 0.0001) (Table 3, Figure 4). 

**Table 3:** Measurements done from control and TOS patients.

	Control patients(n = 32)(interscalene triangle)(mean ± SD)	TOS patients(n = 20)(interscalene triangle)(mean ± SD)	p value
Age	56.45 ± 11.96	44.38 ± 7.16	0.14
Sex (M/F)	9/7	8/10	
Interscalene triangle base length (mm)	17.10 ± 5.45	12.49 ± 0.78	<0.0001
Interscalene triangle height (mm)	47.69 ± 6.30	35.46 ± 4.13	<0.0001
Interscalene space volume by formula (cm³)	4.30 ± 1.85	2.05 ± 0.32	<0.0001

**Table 4:** The measurements of normal and abnormal thoracic outlet region in TOS patients (two patients had bilateral TOS).

	TOS patientsnormal side (interscalene triangle)(n = 16)(mean ± SD)	TOS patientsabnormal side(interscalene triangle)(n = 20)(mean ± SD)	p value
Interscalene triangle base length (mm)	17.43 ± 4.62	12.49 ± 0.78	0.001
Interscalene triangle height (mm)	42.36 ± 5.22	35.46 ± 4.13	0.001
Interscalene space volume by formula (cm³)	4.09 ± 1.11	2.05 ± 0.32	0.001

### 3.3. Surgical treatment-follow up 

All TOS patients underwent trans-axillary approach for scalenectomy, first rib and fibrous bands resection. There was no major complication during surgery. Two patients had pneumothorax at the surgery, and they were treated with a chest tube. The follow-up period ranged from 18–36 months (mean, 24.3 ± 9.6 months). The success of surgical therapy of TOS was defined as complete relief of symptoms postoperatively, assessed by patient-directed outcome questionnaire. According to this questionnaire 14 of 18 patients obtained excellent or good results. The measurements of thoracic outlet region were even smaller in these patients (Table 5). The other 4 patients who had neurologic TOS achieved fair results. In these 4 patient’s volumetric measurements of abnormal sides (1.97 ± 1.03 cm^3^) were close to the healthy sides (2.02 ± 0.68 cm^3^). 

**Table 5:** The measurements of normal and abnormal thoracic outlet region in TOS patients who had achieved excellent or good results.

	TOS patientsnormal side (interscalene triangle)(n = 12)(mean ± SD)	TOS patientsabnormal side(interscalene triangle)(n = 16)(mean ± SD)	p value
Interscalene triangle base length (mm)	17.23 ± 3.72	11.29 ± 0.36	0.001
Interscalene triangle height (mm)	41.16 ± 4.18	33.16 ± 3.12	0.001
Interscalene space volume by formula (cm³)	4.09 ± 1.11	1.99 ± 0.22	0.001

## 4. Discussion

Thoracic outlet syndrome is one of the most hardly diagnosed and treated entrapment neuropathies in humankind. There is a controversy among clinicians regarding its diagnostic criteria and optimal treatment[7,8]. Within the thoracic outlet neurovascular compression typically arise in two regions: the interscalene triangle, and the costa-clavicular space. The interscalene triangle is formed by anterior scalene, middle scalene, and the first rib^1^. Brachial plexus is the most commonly compressed structure (90%), and this compression is created by several factors like cervical ribs, congenital fibrous bands, and scalene muscle hypertrophy[9–11]. 

The size of the interscalene space is interrelated to the variations of the scalene muscles and bony anomalies[12,13]. The changes in the size of this space are the main reason for the symptoms and signs of TOS[14]. There are few studies about the dimensions of the interscalene space[15,16]. But to the best of our knowledge there is no study about the volumetric measurement of this space in English written literature. In this study we have found that interscalene space volume in TOS patients were significantly narrower than the control patients. 

There are not universally dependable and proper diagnostic tests for TOS. The tests used for diagnosis such as electromyography and Doppler ultrasonography do not identify whether the patient has TOS or should have surgery. Electromyography is especially used to determine neurogenic TOS but the benefit in the diagnosis of TOS is controversial [17,18]. Also, clinician should exclude the other potential diseases with the same symptoms. However, diagnosis may not be reliable[19]. Diagnosis of neurogenic TOS is considered as a clinical diagnosis that is primarily based on history and physical examination. In this study, we have found that the volume of interscalene space were smaller than the normal control group, additionally patients with ipsilateral TOS were found to have slightly smaller interscalene space when compared with the contralateral unaffected side. Especially this volumetric measurement technique could help clinicians in diagnosis of suspected neurogenic TOS. 

To assess the outcome of surgery for TOS is difficult, because there are no sufficient tests to compare pre- and post-operative status of the patients[20]. In the follow-up studies, 65% of patients show good results after surgery. Poor outcomes are generally associated with misdiagnosis and incomplete surgery[21]. During the surgery for TOS various complications may develop such as pneumothorax, hematoma, pleural effusion, and neuro-vascular damage[22,23]. Because of these complications a surgical procedure should be offered to carefully selected patients especially with neurogenic TOS. In this study, 4 patients who had neurogenic TOS and achieved fair results, were reassessed, it was seen that the volumetric measurements of abnormal sides were close to the healthy sides. In this way volumetric measurement of the interscalene space could be valuable in selecting patients with neurogenic TOS before planning surgical steps.

There are some limitations of this study. Firstly, the measurements were all collected from preserved cadavers and this can affect the rigidity of muscular structures and consequently volumetric measurements. But measurements from CT sections also have done in control and TOS patients. The aim of the cadaveric volume measurements was to obtain the real anatomic information to compare the volumetric measurements of interscalene space by two different methods and to verify the volume calculating formula. Secondly, the specimen numbers in study groups for cadaveric measurements and for TOS patients were small so it is unfortunately not suitable to define a cut-off value or ROC curves for volumetric measurements. But this project is still going on and, in the future, we will define a cut-off value when the number of the patients is sufficient. 

In this study we have found that interscalene space volume was smaller in TOS patients and this volume could be calculated by the measurements done from the CT sections with a simple formula. In this respect, volumetric measurements from CT sections could help clinicians in diagnosis of TOS especially in suspected cases. Additionally, this volumetric measurement has a potential value for selecting patients for surgery.

## Informed consent

This study was approved by the Ufuk University Hospital ethics review board and consent of all patients has been obtained. Ethic review board number is 02062015-3.

## References

[ref1] (1996). Thoracic outlet compression syndrome. Orthopedic Clinics of North America.

[ref2] (2010). Thoracic outlet syndrome: a controversial clinical condition. Part I: anatomy and clinical examination/diagnosis. Journal of Manual and Manipulative Therapy.

[ref3] (1999). Thoracic outlet syndromes. Neurologic Clinics.

[ref4] (1996). Thoracic outlet syndrome: fact or fancy? A review of 409 consecutive patients who underwent operation. Canadian Journal of Surgery.

[ref5] (2001). Outcomes after surgery for thoracic outlet syndrome. Journal of Vascular Surgery.

[ref6] (1985). Stability of some soluble alginate solutions. Biomaterials.

[ref7] (2002). Thoracic outlet syndrome. Current Problems in Surgery.

[ref8] (2001). Porter JM. Long-term functional outcome of neurogenic thoracic outlet syndrome in surgically and conservatively treated patients. Journal of Vascular Surgery.

[ref9] (2007). First rib resection in thoracic outlet syndrome. Journal of Hand Surgery (Am).

[ref10] (2003). Transaxillary approach in thoracic outlet syndrome: the importance of resection of the first rib. European Journal of Cardiothoracic Surgery.

[ref11] (2004). Thoracic outlet syndrome. Neurosurgery.

[ref12] (1997). Scalene muscles and brachial plexus: anatomical variations and their clinical significance. Clinical Anatomy.

[ref13] (2001). Anatomical variations of the scalene triangle: dissection of 10 cadavers. Journal of Orthopaedic and Sports Physical Therapy.

[ref14] (2006). Anatomy of inter-scalane triangle and its role in thoracic outlet syndrome. Journal of the Anatomical Society of India.

[ref15] (2012). Descriptive anatomy of the interscalene triangle and the costoclavicular space and their relationship to thoracic outlet syndrome: a study of 60 cadavers. Journal of Manipulative and Physiological Therapeutics.

[ref16] (2018). The importance of costoclavicular space on possible compression of the subclavian artery in the thoracic outlet region: a radio-anatomical study. Interactive Cardiovascular and Thoracic Surgery.

[ref17] (1993). SE Mckinnon, GA Patterson. The Journal of Hand Surgery.

[ref18] (1998). Thoracic outlet syndrome. Journal of Shoulder and Elbow Surgery.

[ref19] (1996). Thoracic outlet compromise.

[ref20] (1986). Surgery for thoracic outlet syndrome may be hazardous to your health. Muscle and Nerve.

[ref21] (1996). Results of the surgical treatment for thoracic outlet syndrome. Seminars in Thoracic and Cardiovascular Surgery.

[ref22] (2011). Early results of a highly selective algorithm for surgery on patients with neurogenic thoracic outlet syndrome. Journal of Vascular Surgery.

[ref23] (2009). Surgical intervention for thoracic outlet syndrome improves patient’s quality of life. Journal of Vascular Surgery.

